# Supplementary material for: FADS1-arachidonic acid axis enhances arachidonic acid metabolism by altering intestinal microecology in colorectal cancer
Source: Nat Commun. 2023 Apr 11;14:2042. doi: 10.1038/s41467-023-37590-x (PMC10090135; doi:10.1038/s41467-023-37590-x)
Supplement: Supplementary file 1 — Supplementary Information [file 41467_2023_37590_MOESM1_ESM.pdf]

FADS1-arachidonic acid axis enhances arachidonic acid metabolism by altering intestinal microecology in colorectal cancer

Supplementary figure and figure legends

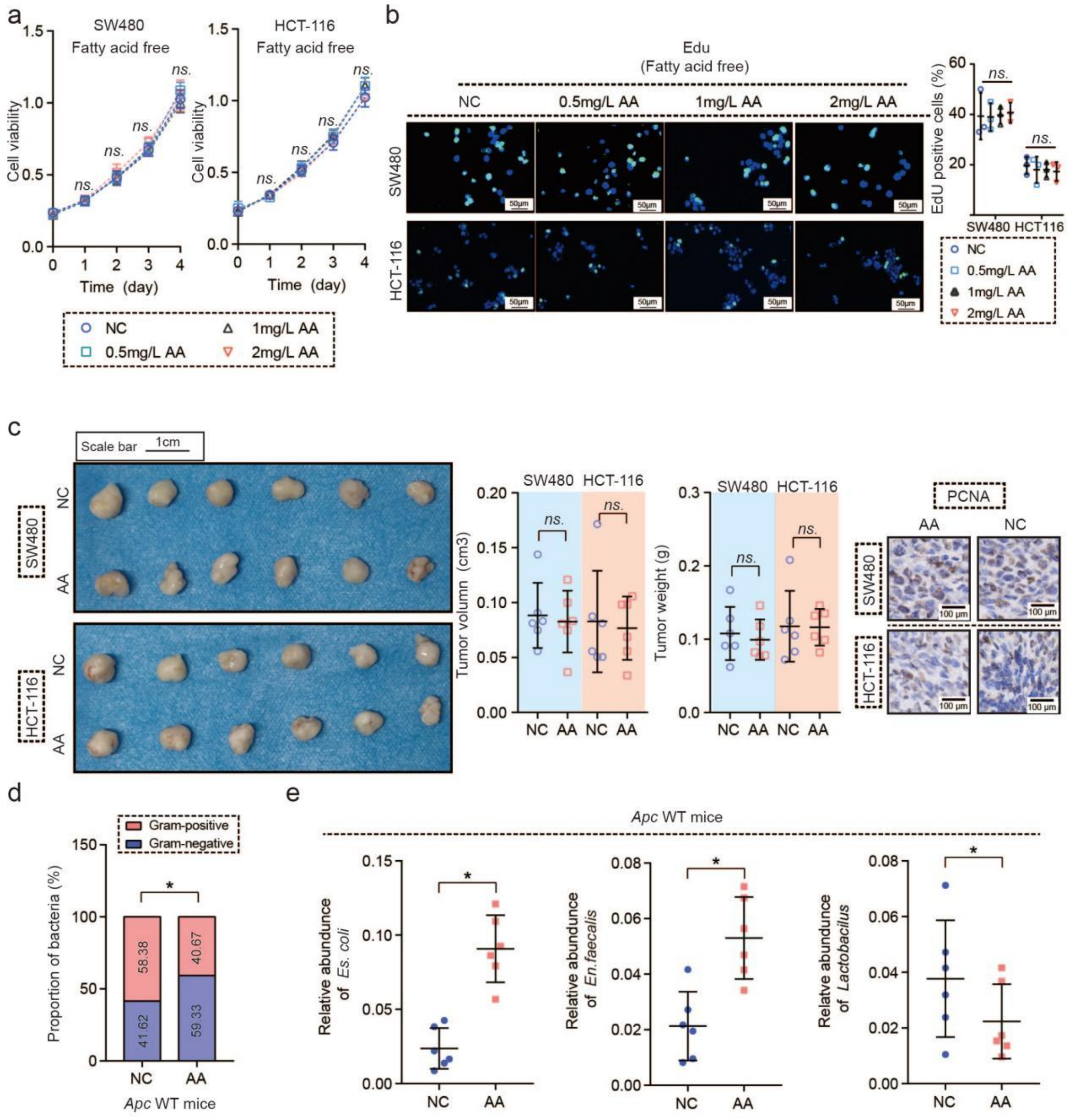

Supplementary Fig 1. AA had no effect on cell proliferation *in vitro* and subcutaneous tumor. a. The viability of SW480 and HCT116 cells treated with different concentration (NC, 0.5mg/L, 1mg/L, 2mg/L) of AA, cultured with fatty acid free FBS, and

analyzed by CCK-8 (n=5 samples per group; means ± s.d., one-way repeated-measures ANOVA, n=3 biological replicates). ns indicates no statistical significance. b. EdU assay for SW480 and HCT116 cells treated with different concentrations (NC, 0.5mg/L, 1mg/L, 2mg/L) of AA, cultured with fatty acid free FBS (n=5 samples per group, 3 fields assessed per sample, means ± s.d., two-tailed unpaired *t* test). ns indicates no statistical significance. Scale bars, 50 µm. c. Representative image of the subcutaneous tumors and PCNA expression in tumor tissues with AA (2mg/L) treatment via intratumor injection in SW480 and HCT116 cells (n=6 mice per group, means ± s.d., two-tailed unpaired *t* test). Tumor weight and volume (length*width^2/2) were calculated. ns indicates no statistical significance. Scale bars, 100 µm. d. Ratio of gram-negative bacteria to gram-positive bacteria in the stool of *Apc* WT mice with NC and AA feeding (n=6 mice per group, *Chi* square test, n=3 technical replicates). *p=0.038. e. Relative abundance of *Es. coli, En. faecalis* and *Lactobacillus* in the stool of *Apc* WT mice with NC and AA feeding (n=6 mice per group, means ± s.d., two-tailed unpaired *t* test, n=3 technical replicates). *p=0.047, 0.045, 0.039. Source data are provided in the Source Data file.

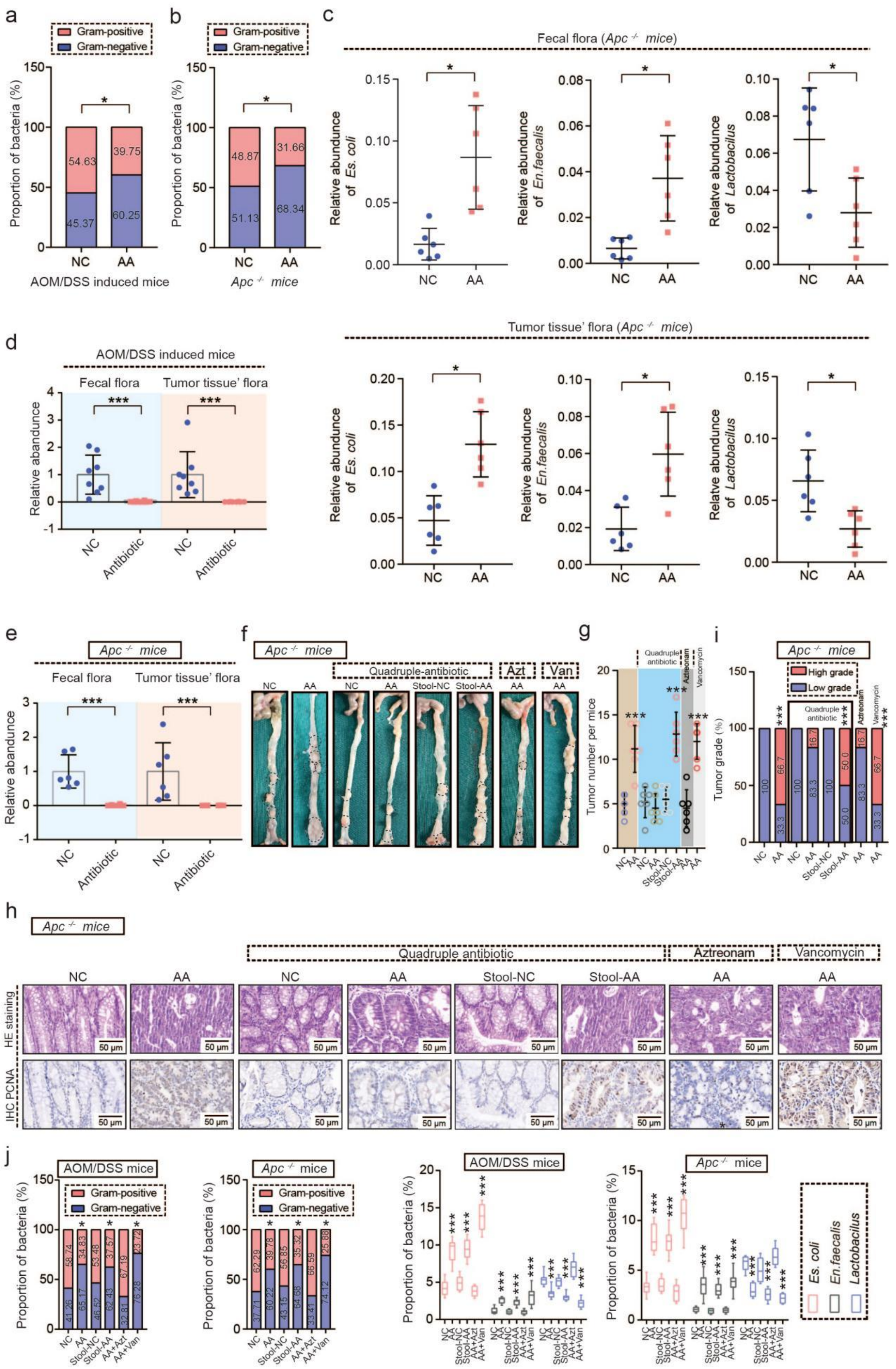

Supplementary Fig 2. Gut microbes mediate the

tumor-promoting role of AA in CRC process. a. Ratio of gram-negative bacteria and gram-positive bacteria in the stool of AOM/DSS induced mice with NC or AA feeding (n=6 mice per group, *Chi* square test, n=3 biological replicates). *p=0.034. b. Ratio of gram-negative bacteria and gram-positive bacteria in intestinal specific *Apc* $^{-/-}$ mice with NC or AA feeding (n=6 mice per group, *Chi* square test, n=3 biological replicates). *p=0.014. c. Relative abundance of *Es. coli, En. faecalis* and *Lactobacillus* in the stool and colon tissue of intestine-specific *Apc*$^{-/-}$ mice with NC and AA feeding (n=6 mice per group, means ± s.d., two-tailed unpaired t test, n=3 biological replicates). *p=0.016, 0.037, 0.048. d. Gut microbial elimination in AOM/DSS induced mice (n=6 mice per group, means ± s.d., two-tailed unpaired t test, n=3 biological replicates). ***$p < 0.001$. e. Gut microbial elimination in intestine-specific *Apc*$^{-/-}$ mice (n=6 mice per group, means ± s.d., two-tailed unpaired t test, n=3 biological replicates). ***$p < 0.001$. f. Tumor images of the intestine-specific *Apc*$^{-/-}$ mice with NC or AA feeding under different antibiotics treatment (Quadruple-antibiotics, 0.2g/L of aztreonam, 0.1g/L of vancomycin) (n=6 mice per group). Black circles showed tumor region. g. Tumor number of the intestine-specific *Apc*$^{-/-}$ mice with NC or AA feeding under different antibiotics treatment (n=6 mice per group, means ± s.d., two-tailed unpaired t test). ***p <

0.001 (compared with NC group). h. HE staining and PCNA expression in the tumor tissue of the intestine-specific *Apc*$^{-/-}$ mice with NC or AA feeding under different antibiotics treatment (n=6 mice per group). Scale bars, 50 μm. i. Tumor grades of the intestine-specific *Apc*$^{-/-}$ mice with NC or AA feeding under different antibiotics treatment (n=6 mice per group,3 fields assessed per sample, *Chi* square test). ***$p < 0.001$ (compared with NC group). j. Gut microbes alteration in AOM/DSS and intestine-specific *Apc*$^{-/-}$ mice with the fecal transplantation and different antibiotics (n=6 mice per group, *Chi* square test, Box plots: Min to Max; n=3 biological replicates). *$p =0.001, 0.003, <0.001, 0.002, 0.001, <0.001$, ***$p < 0.001$ (compared with NC group). Source data are provided in the Source Data file.

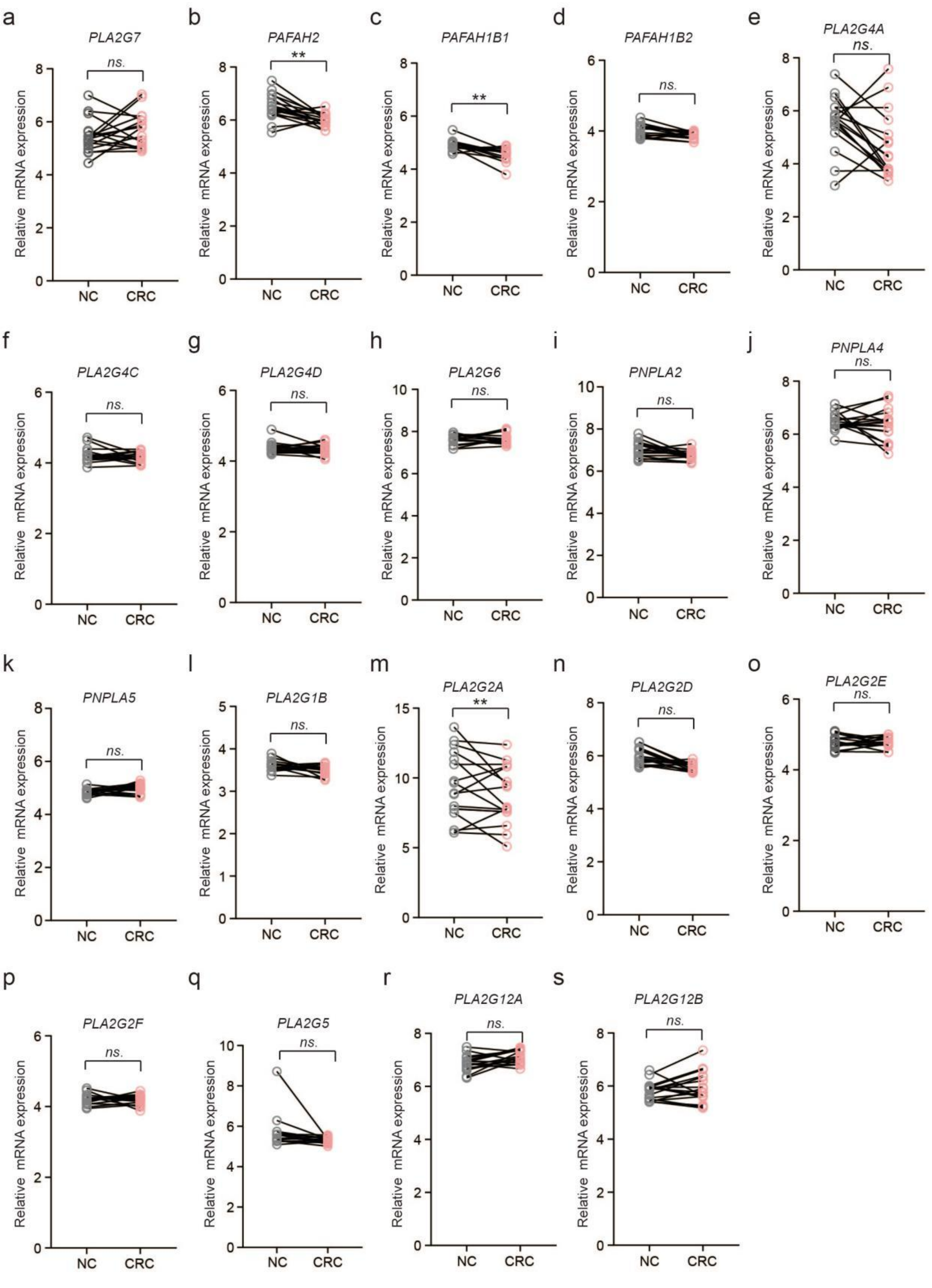

Supplementary Fig 3. **The mRNA expression of PLA enzymes in GDS4382.** (a-s) The mRNA expression of *PLA2G7*, *PAFAH2*, *PAFAH1B1*, *PAFAH1B2*, *PLA2G4A*, *PLA2G4C*, *PLA2G4D*, *PLA2G6*,

PNPLA2, *PNPLA4*, *PNPLA5*, *PLA2G1B*, *PLA2G2A*, *PLA2G2D*, *PLA2G2E*, *PLA2G2F*, *PLA2G5*, *PLA2G12A*, *PLA2G12B* in 17 cases of paired NC and CRC samples. Measurement data were presented as the mean ± s.d.. The two-tailed paired student's *t*-test was used for statistical analysis. ***p=0.002, 0.003, 0.008*, ns represents no statistical significance. Source data are provided in the Source Data file.

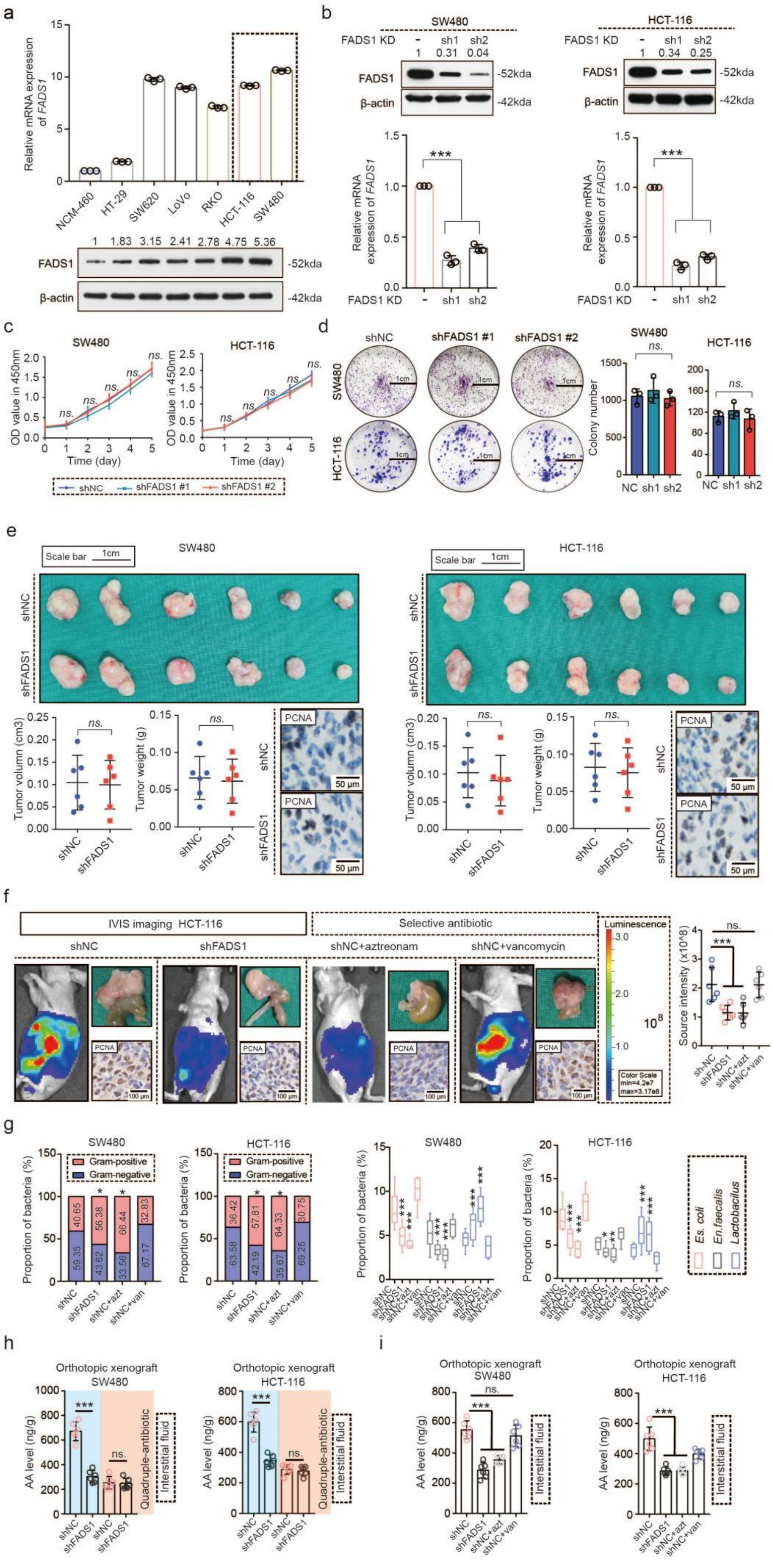
a
b
c
d
e
f
g
h
i
SW480
HCT-116
FADS1 KD
FADS1
β-actin
-52kda
-42kda
Relative mRNA expression of FADS1
NCM-460
HT-29
SW620
LoVo
RKO
1 1.83 3.15 2.41 2.78 4.75 5.36
OD value in 450nm
Time (day)
shNC
shFADS1 #1
shFADS1 #2
Colony number
Scale bar 1cm
Tumor volumn (cm3)
Tumor weight (g)
PCNA
50 μm
IVIS imaging HCT-116
Selective antibiotic
shNC+aztreonam
shNC+vancomycin
Luminescence
Source intensity (x10^8)
Proportion of bacteria (%)
Gram-positive
Gram-negative
Es. coli
En.faecalis
Lactobacilus
Orthotopic xenograft
AA level (ng/g)
Quadruple-antibiotic
Interstitial fluid

Supplementary Fig 4. FADS1 knockdown did not affect the proliferation of CRC cells. a. The expression of FADS1 in colonic epithelial and CRC cell lines. The expression of FADS1 was detected by q-PCR and western blot (n=6 mice per group, means ± s.d., n=3 biological replicates). b. *FADS1* knockdown in SW480 and HCT116 cells by shRNAs. The efficiency of *FADS1* knockdown was detected (n=6 mice per group, means ± s.d., two-tailed unpaired *t* test, n=3 biological replicates). ***$p < 0.001$. c. The viability of SW480 and HCT116 cells transfected with sh*FADS1* or shNC (n=5 samples per group; means ± s.d., one-way repeated-measures ANOVA, n=3 biological replicates). ns represents no statistical significance. d. Colony formation ability of SW480 and HCT116 cells transfected with sh*FADS1* or shNC (n=3 per group, means ± s.d., two-tailed unpaired *t* test, n=3 biological replicates). ns represents no statistical significance. e. The subcutaneous tumors and PCNA expression in the tumor tissues injected with shNC and sh*FADS1* SW480 or HCT116 cells (n=6 mice per group, means ± s.d., two-tailed unpaired *t* test). ns represents no statistical significance. Scale bars, 50 μm. f. IVIS imaging of the orthotopic tumor injected by shNC and sh*FADS1* $HCT116^{Luc}$ cells (n=6 mice per group, means ± s.d., two-tailed unpaired *t* test), gut microbes were deleted by aztreonam or vancomycin treatment. Scale colour

bar: 4.20×10^7-3.17×10^8. ***p < 0.001. ns represents no statistical significance. g. Ratio of gram-negative and gram-positive bacteria in the orthotopic tumors in shNC, sh*FADS1*, shNC+aztreonam, shNC+vancomycin groups (n=6 mice per group, *Chi* square test, Box plots: Min to Max; n=3 biological replicates). *p=0.034, 0.001, 0.002, <0.001, 0.017, **p=0.006, ***p<0.001 (compared with shNC). h. AA levels in the interstitial fluid of shNC, *shFADS1*, shNC+quadruple-antibiotic and sh*FADS1*+quadruple-antibiotic orthotopic tumors (n=6 mice per group, means ± s.d., two-tailed unpaired *t* test, n=3 technical replicates). ***p<0.001. ns represents no statistical significance. i. AA levels in the interstitial fluid of shNC, sh*FADS1*, shNC+aztreonam, shNC+vancomycin orthotopic tumors (n=6 mice per group, means ± s.d., two-tailed unpaired *t* test, n=3 technical replicates). ***p<0.001. ns represents no statistical significance. Source data are provided in the Source Data file.

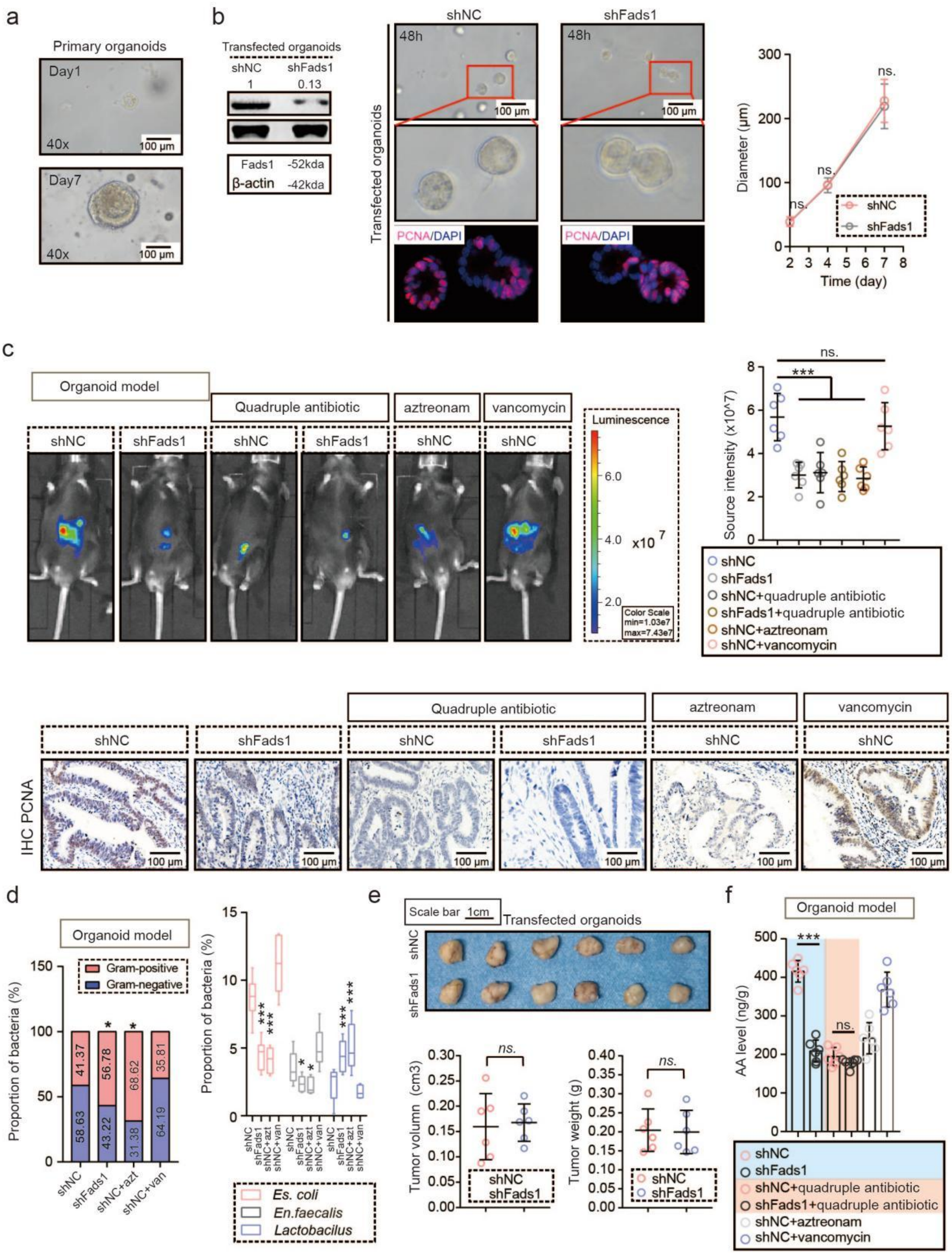

Supplementary Fig 5. The tumor-promoting role of FADS1 in CRC organoid model. a. Establishment of CRC organoid model by AOM/DSS induced mice. Scale bars, 100 μm. b. Transfected organoids with shNC and sh*Fads1* by shRNAs. The efficiency of Fads1 knockdown was detected by western blot (n=3 biological

replicates). Scale bars, 100 μm. The diameter of the organoid was detected at the different time point (2 day, 4 day, 7 day) (n=5 samples per group, 3 fields assessed per sample, means ± s.d., two-tailed unpaired *t* test, n=3 biological replicates). ns represents no statistical significance. c. IVIS imaging of the orthotopic tumor injected by shNC and sh*Fads1* organoid cells under different antibiotics treatment (Quadruple-antibiotics, 0.2g/L of aztreonam, 0.1g/L of vancomycin) (n=6 mice per group, means ± s.d., two-tailed unpaired *t* test). Luminescence was analyzed and representative images of PCNA expression in shNC and sh*Fads1* organoid tissues. ***$p<0.001$. ns represents no statistical significance. Scale bars, 100 μm. d. Ratio of gram-negative and gram-positive bacteria in the orthotopic tumors of shNC, sh*Fads1*, shNC+aztreonam, shNC+vancomycin groups (n=6 mice per group, *Chi* square test, Box plots: Min to Max; n=3 biological replicates). *$p=0.024$, <0.001, 0.035, 0.018, ***$p<0.001$ (compared with shNC). e. Representative image of the subcutaneous tumors injected by shNC and sh*Fads1* organoid cells (n=6 mice per group, means ± s.d., two-tailed unpaired *t* test,). Tumor weight and volume (length*width^2/2) were calculated. ns represents no statistical significance. f. AA levels in the interstitial fluid of shNC, sh*Fads1*, shNC+quadruple-antibiotic, shFads1+quadruple-antibiotic, shNC+aztreonam, shNC+vancomycin

orthotopic tumors (n=6 mice per group, means ± s.d., two-tailed unpaired *t* test, n=3 technical replicates). ***p<0.001. ns represents no statistical significance. Source data are provided in the Source Data file.

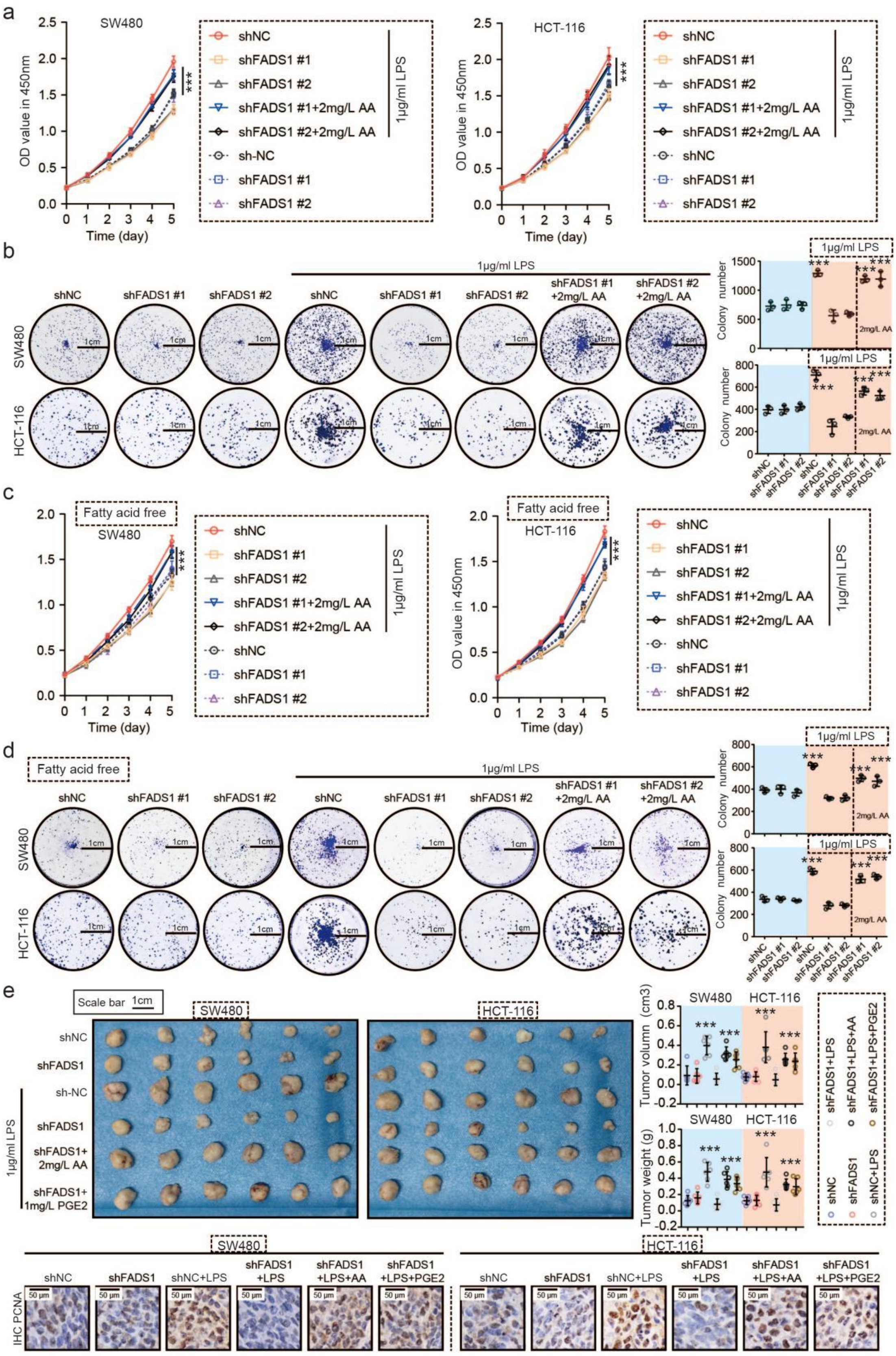
a
SW480
HCT-116
OD value in 450nm
Time (day)
shNC
shFADS1 #1
shFADS1 #2
shFADS1 #1+2mg/L AA
shFADS1 #2+2mg/L AA
sh-NC
1μg/ml LPS
b
1μg/ml LPS
shNC
shFADS1 #1
shFADS1 #2
shFADS1 #1 +2mg/L AA
shFADS1 #2 +2mg/L AA
Colony number
2mg/L AA
c
Fatty acid free
d
e
Scale bar 1cm
shFADS1
shFADS1+ 2mg/L AA
shFADS1+ 1mg/L PGE2
Tumor volumn (cm3)
Tumor weight (g)
shNC+LPS
shFADS1+LPS
shFADS1+LPS+AA
shFADS1+LPS+PGE2
IHC PCNA
50 μm

Supplementary Fig 6. **FADS1 knockdown decreased the CRC cell proliferation upon LPS activation.** a. The viability of SW480 and HCT116 cells transfected with sh*FADS1* or shNC, under activation of 1μg/ml of LPS and 2mg/L of AA treatment, as analyzed with CCK-8 assay (n=5 samples per group; means ± s.d., one-way repeated-measures ANOVA, n=3 biological replicate). ***$p<0.001$. b. Colony formation ability of SW480 and HCT116 cells transfected with sh*FADS1* or shNC, under activation of 1μg/ml of LPS and 2mg/L of AA treatment, as analyzed with colony formation assay (n=3 per group, means ± s.d., two-tailed unpaired *t* test, n=3 biological replicates). ***$p<0.001$ (compared with shNC group). c. The viability of SW480 and HCT116 cells transfected with sh*FADS1* or shNC, under activation of 1μg/ml of LPS and 2mg/L of AA treatment, cultured with fatty acid free FBS (n=5 samples per group; means ± s.d., one-way repeated-measures ANOVA, n=3 biological replicate). ***$p<0.001$. d. Colony formation ability of SW480 and HCT116 cells transfected with sh*FADS1* or shNC, under activation of 1μg/ml of LPS and 2mg/L of AA treatment, cultured with fatty acid free FBS (n=3 per group, means ± s.d., two-tailed unpaired *t* test, n=3 biological replicates). ***$p<0.001$(compared with shNC group). Scale bars, 1cm. e. Representative image of the subcutaneous tumors injected with shNC and sh*FADS1* SW480 or

HCT116 cells, under activation of 1μg/ml of LPS and 2mg/L of AA or 1mg/L of PGE2 treatment by intratumoral injection (n=6 mice per group, means ± s.d., two-tailed unpaired *t* test). Tumor weight and volume (length*width^2/2) were calculated. ***p<0.001 (compared with shNC group). Representative images of PCNA expression in the subcutaneous tumors was showed. Scale bars, 50 μm. Source data are provided in the Source Data file.

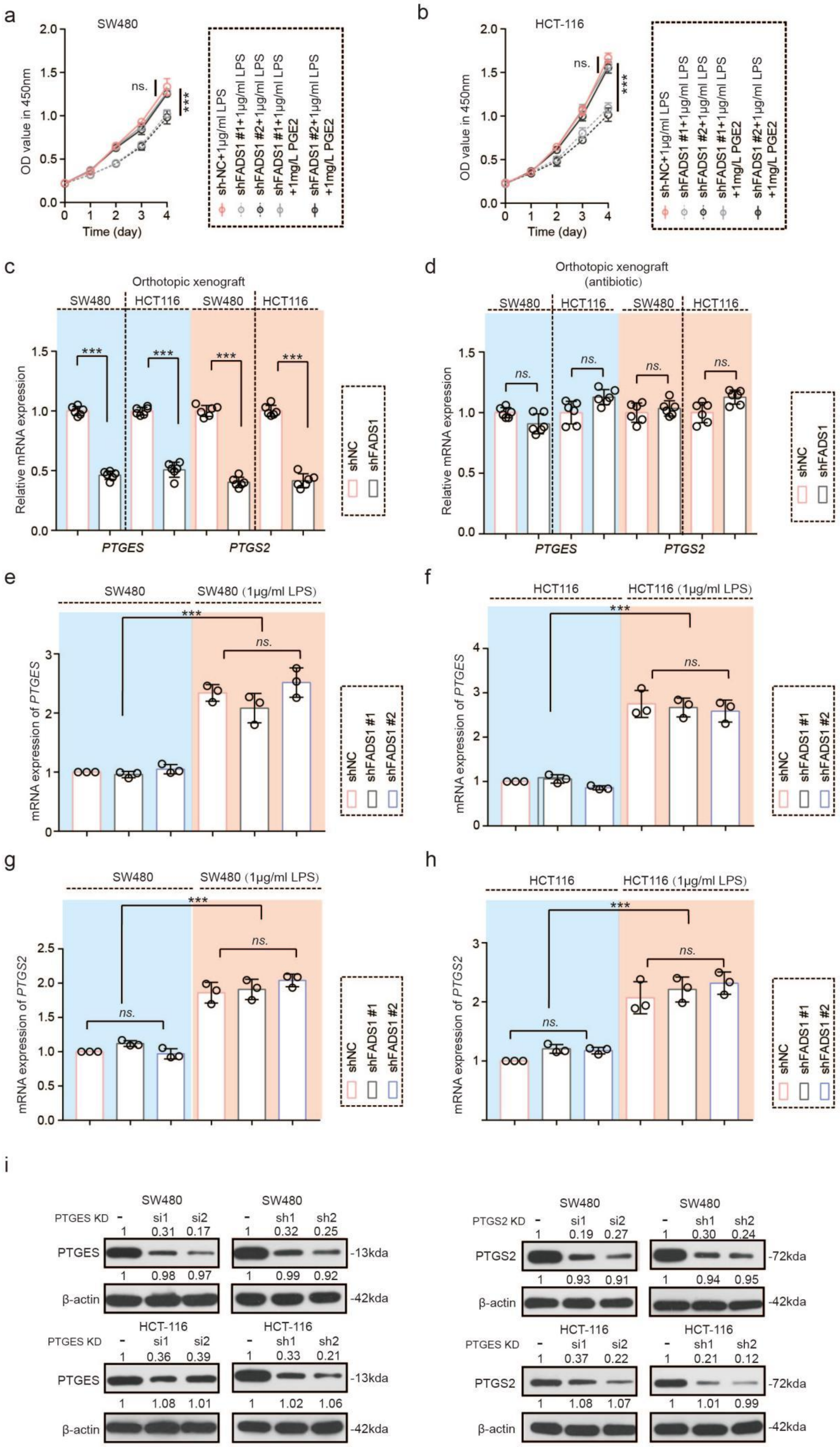
a
SW480
OD value in 450nm
Time (day)
ns.
***
sh-NC+1μg/ml LPS
shFADS1 #1+1μg/ml LPS
shFADS1 #2+1μg/ml LPS
shFADS1 #1+1μg/ml LPS +1mg/L PGE2
shFADS1 #2+1μg/ml LPS +1mg/L PGE2
b
HCT-116
c
Orthotopic xenograft
SW480
HCT116
Relative mRNA expression
PTGES
PTGS2
shNC
shFADS1
d
Orthotopic xenograft (antibiotic)
e
SW480 (1μg/ml LPS)
mRNA expression of PTGES
shFADS1 #1
shFADS1 #2
f
HCT116 (1μg/ml LPS)
g
mRNA expression of PTGS2
h
i
PTGES KD
PTGS2 KD
si1
si2
sh1
sh2
PTGES
PTGS2
β-actin
-13kda
-42kda
-72kda

Supplementary Fig 7. LPS activation promotes the mRNA expression of *PTGES* and *PTGS2*. a. The viability of SW480 cells transfected with sh*FADS1* or shNC, under activation of 1μg/ml of LPS and 1mg/L of PGE2 treatment (n=5 samples per group; means ± s.d., one-way repeated-measures ANOVA, n=3 biological replicate). ***$p<0.001$. ns represents no statistical significance. b. The viability of HCT116 cells transfected with sh*FADS1* or shNC, under activation of 1μg/ml of LPS and 1mg/L of PGE2 treatment (n=5 samples per group; means ± s.d., one-way repeated-measures ANOVA, n=3 biological replicate). ***$p<0.001$. ns represents no statistical significance. c. The mRNA expressions of *PTGES* and *PTGS2* in the tumor tissues of the orthotopic model injected with SW480 and HCT116 cells transfected with sh*FADS1* or shNC (n=6 samples per group, means ± s.d., two-tailed unpaired t test, n=3 technical replicates). ***$p<0.001$. d. The mRNA expressions of *PTGES* and *PTGS2* in the orthotopic tumor injected by SW480 and HCT116 cells transfected with sh*FADS*1 or shNC (n=6 samples per group, means ± s.d., two-tailed unpaired t test, n=3 technical replicates), gut microbes were deleted by antibiotic treatment. ns represents no statistical significance. e and f. The mRNA expression of *PTGES* in SW480 cells transfected with sh*FADS1* or shNC, with or without activation of 1μg/ml of LPS (n=3 samples per group,

means ± s.d., two-tailed unpaired *t* test, n=3 biological replicates). ***p<0.001. ns represents no statistical significance. g. The mRNA expression of *PTGS2* in SW480 cells transfected with sh*FADS1* or shNC, with or without activation of 1μg/ml of LPS (n=3 samples per group, means ± s.d., two-tailed unpaired *t* test, n=3 biological replicates). ***p<0.001. ns represents no statistical significance. h. The mRNA expression of *PTGS2* in HCT116 cells transfected with sh*FADS1* or shNC, with or without activation of 1μg/ml LPS (n=3 samples per group, means ± s.d., two-tailed unpaired *t* test, n=3 biological replicates). ***p<0.001. ns represents no statistical significance. i. *PTGES* and *PTGS2* knockdown in SW480 and HCT116 cells by siRNAs and shRNAs, detected by western blot (n=3 biological replicates). Source data are provided in the Source Data file.

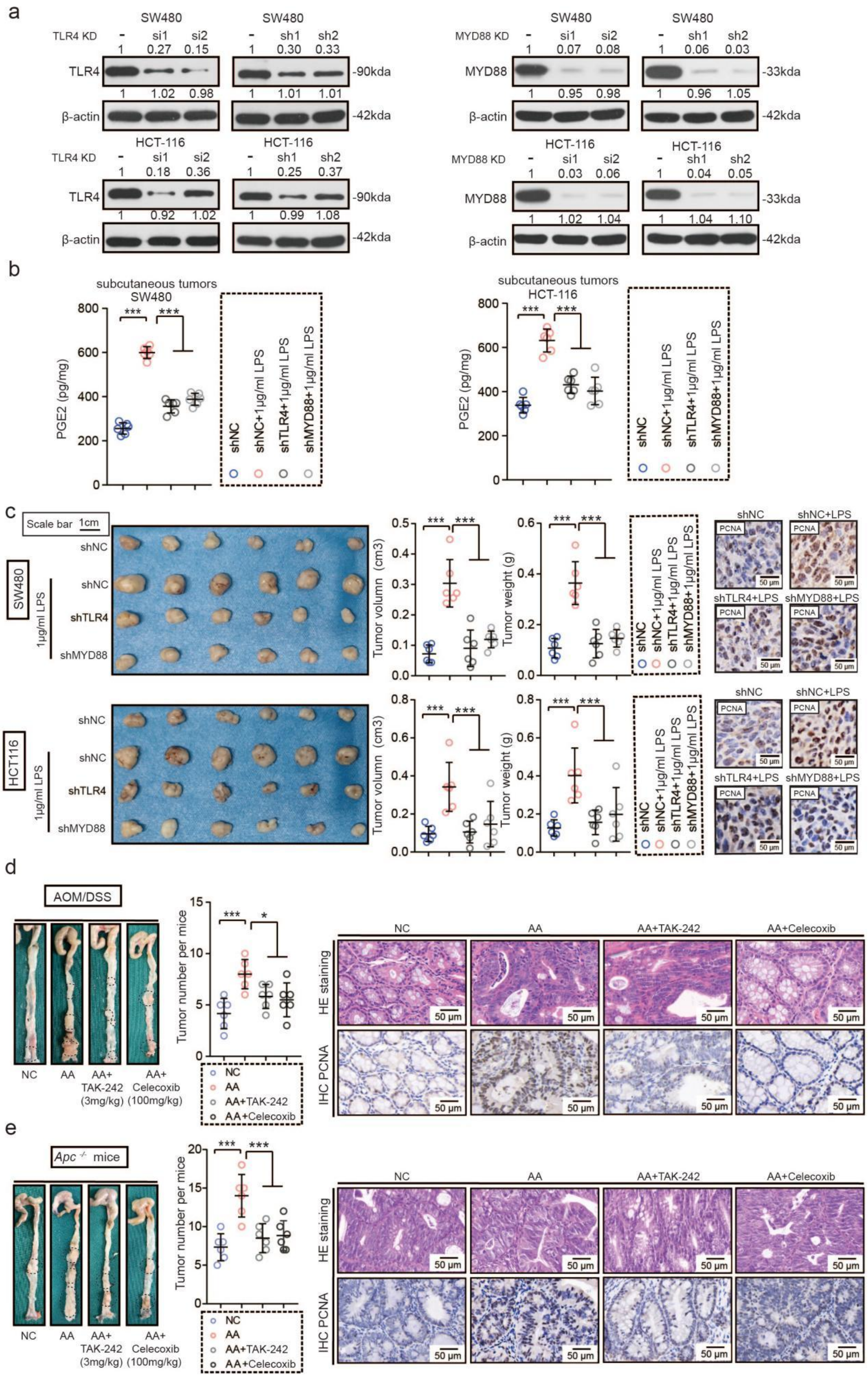

283 **Supplementary Fig 8. Inhibition of TLR4/MYD88 pathway**

impaired LPS-induced cell proliferation of CRC tumor. a. *TLR4* and *MYD88* knockdown in SW480 and HCT116 cells by siRNAs and shRNAs. The efficiency of *TLR4* and *MYD8*8 knockdown was detected by western blot (n=3 biological replicates). b. PGE2 levels in the subcutaneous tumors injected with sh*TLR4*, sh*MYD88* and shNC SW480 or HCT-116 cells, under activation of 1μg/ml of LPS. ***p<0.001. (n=6 samples per group, means ± s.d., two-tailed unpaired t test, n=3 technical replicates). c. Representative image of the subcutaneous tumors injected with sh*TLR*4, sh*MYD88* and shNC SW480 or HCT-116 cells, under activation of 1μg/ml of LPS by intratumoral injection (n=6 mice per group, means ± s.d., two-tailed unpaired t test). Tumor weight and volume (length*width^2/2) were calculated. ***p<0.001. Representative images of PCNA expression in the subcutaneous tumors was showed. Scale bars, 50 μm. d. Representative image of AOM/DSS model with NC and AA feeding, treated by TLR4 inhibitor (TAK-242, 3mg/kg, intraperitoneal injection) and PTGS2 inhibitor (Celecoxib, 100mg/kg, oral treatment) (n=6 mice per group, means ± s.d., two-tailed unpaired t test). Black circles showed the tumor region. *p=0.036, ***p<0.001. Representative images of PCNA expression in the tumors was showed. Scale bars, 50 μm. e. Representative image of the intestine-specific $Apc^{-/-}$ model with NC and AA feeding, treated by

TLR4 inhibitor (TAK-242, 3mg/kg, intraperitoneal injection) and PTGS2 inhibitor (Celecoxib, 100mg/kg, oral treatment). Black circles showed the tumor region (n=6 mice per group, means ± s.d., two-tailed unpaired *t* test). ***p<0.001. Representative images of PCNA expression in the tumors was showed. Scale bars, 50 μm. (n=6 mice per group). Source data are provided in the Source Data file.

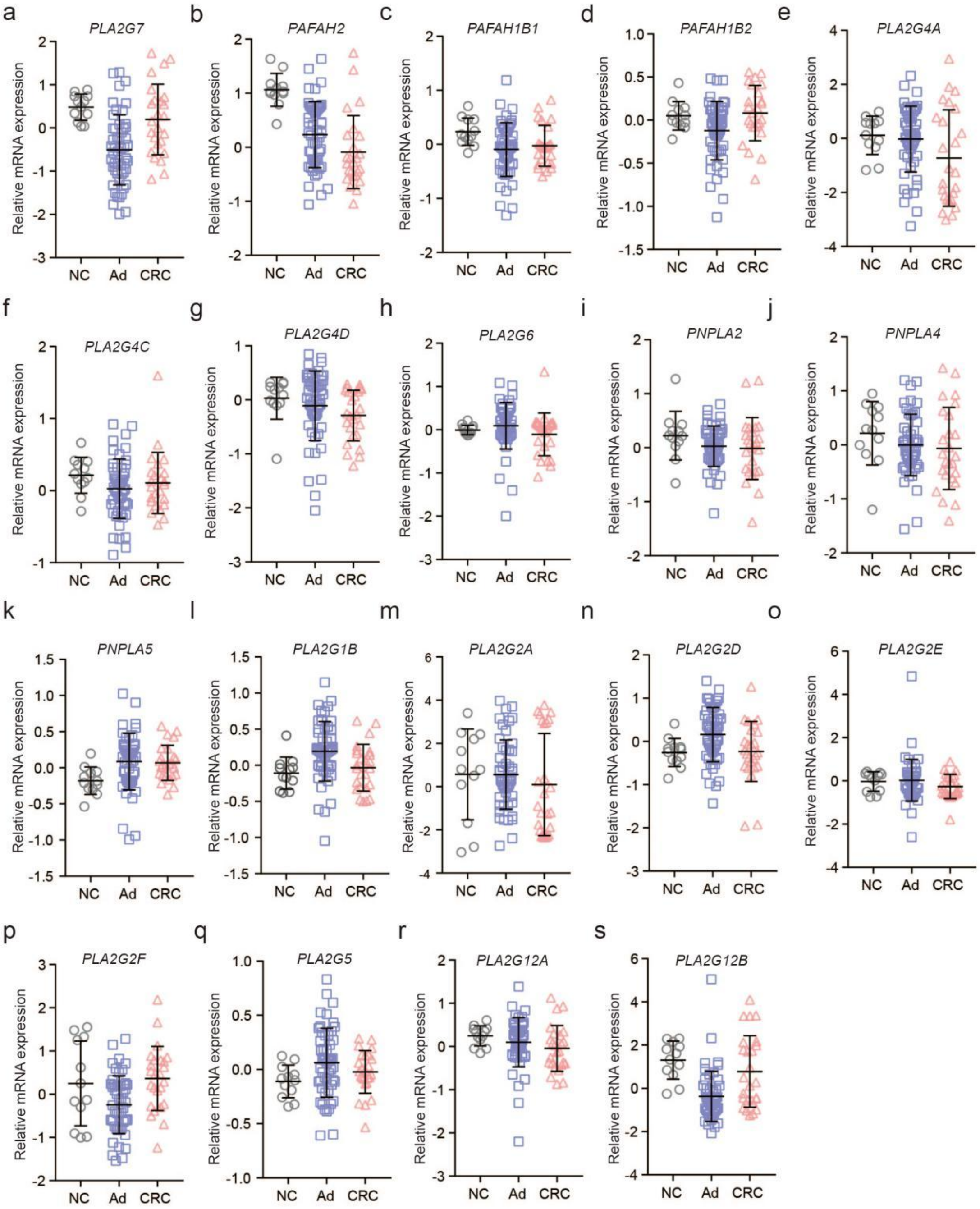

Supplementary Figure 9. The mRNA expression of PLA enzymes in GSE41657. (a-s) The mRNA expression of *PLA2G7*, *PAFAH2*,

PAFAH1B1, *PAFAH1B2*, *PLA2G4A*, *PLA2G4C*, *PLA2G4D*, *PLA2G6*, *PNPLA2*, *PNPLA4*, *PNPLA5*, *PLA2G1B*, *PLA2G2A*, *PLA2G2D*, *PLA2G2E*, *PLA2G2F*, *PLA2G5*, *PLA2G12A*, *PLA2G12B* in NC (n=13 samples), Ad (n=50 samples) and CRC (n=25 samples) samples. Measurement data were presented as the mean ± s.d..

Supplementary Table 1 Interference sequence and primers of target genes used in this study.

		**Sequence**
***TLR4* siNC**	**Sense**	5'-AGGACAGGGUUCUCCCUUCCUCUAU-3'
***TLR4* si1**	**Sense**	5'-AGGCUGACAUUGGCCUCCUUCCUAU-3'
***TLR4* si2**	**Sense**	5'-CCACACCCAAUUGCUUCAUGCUUAA-3'
***MYD88* siNC**	**Sense**	5'-UUCUCCGAACGUGUCACGUTT-3'
***MYD88* si1**	**Sense**	5'-GCCTATCGCTGTTCTTGAA-3'
***MYD88* si2**	**Sense**	5'-GACTGATTCCTATTAAATA-3'
***PTGES* siNC**	**Sense**	5'-UCGUCAUUUCCGUCUCUGGCCUUAA-3'
***PTGES* si1**	**Sense**	5'-GGGACTTGATGTTCCTTCCAGATTG-3'
***PTGES* si2**	**Sense**	5'-CCUUGCCGUUGGCUUUGGAUGUCUU-3'
***PTGS2* siNC**	**Sense**	5'-GAGUAUGUGGUCAUACCGAAUUUCA-3'
***PTGS2* si1**	**Sense**	5'-GAGUUAUGUGUUGACAUCCAGAUCA-3'
***PTGS2* si2**	**Sense**	5'-GAAGCCUUCUCUAACCUCUCCUAUU-3'
***FADS1* shNC**	**Sense**	5'-UCUACUCAUUCGUGUCACCUCUAUU-3'
***FADS1* sh1**	**Sense**	5'-GGGCCTTGTGAAGAAGTATA-3'
***FADS1* sh2**	**Sense**	5'-GCAAAGACCCAGACATCAACA-3'
***TLR4* shNC**	**Sense**	5'-AAGACUACUGUUUCGACUUCUCUAA-3'
***TLR4* sh1**	**Sense**	5'-CACGGCATCTTTACTGGCTTAGTCA-3'
***TLR4* sh2**	**Sense**	5'-CATCTTCACAGAGCTGACTAACTTA-3'
***MYD88* shNC**	**Sense**	5'-TTCTCCGAACGTGTCACGTTT-3'
***MYD88* sh1**	**Sense**	5'-GCACATACAGACTCACATACA-3'
***MYD88* sh2**	**Sense**	5'-GACATGTACTCTCACACACAC-3'
***PTGES* shNC**	**Sense**	5'-GGUUUCGACUAUUUCAACGGCCUUU-3'
***PTGES* sh1**	**Sense**	5'-GGAACGACATGGAGACCATCT-3'
***PTGES* sh2**	**Sense**	5'-GGGCTTCGTCTACTCCTTTCT-3'
***PTGS2* shNC**	**Sense**	5'-AATTCTCCGAACGTGTCACGT-3'
***PTGS2* sh1**	**Sense**	5'-GCAACACTTGAGTGGCTATCA-3'

***PTGS2* sh2**	**Sense**	5'- GCTTTATGCTGAAGCCCTATG-3'
**shNC**	**Sense**	5'-CACAGAATAGAACGCAATTCCAGAT-3'
**Sh*Fads1***	**Sense**	5'-GCACATGCCATACAACCATCA-3'
FADS1	**Forward**	5’-CCAACTGCTTCCGCAAAGAC-3’
	**Reverse**	5’-GCTGGTGGTTGTACGGCATA-3’
18S	**Forward**	5’-TGCGAGTACTCAACACCAACA-3’
	**Reverse**	5’-GCATATCTTCGGCCCACA-3’
TLR4	**Forward**	5’-AGACCTGTCCCTGAACCCTAT-3’
	**Reverse**	5’-CGATGGACTTCTAAACCAGCCA-3’
MYD88	**Forward**	5’-GGCTGCTCTCAACATGCGA-3’
	**Reverse**	5’-CTGTGTCCGCACGTTCAAGA-3’
PTGES	**Forward**	5’-TCCTAACCCTATTGTCGCCTG-3’
	**Reverse**	5’-CGCTTCCCAGAGGATCTGC-3’
PTGS2	**Forward**	5’-TAAGTGCGATTGTACCCGGAC-3’
	**Reverse**	5’-TTTGTAGCCATAGTCAGCATTGT-3’

Supplementary Table 2 Gender of human research participants

Case	Gender	Case	Gender	Case	Gender	Case	Gender	Case	Gender
1	Male	101	Female	201	Male	301	Male	1	Male
2	Female	102	Female	202	Male	302	Male	2	Male
3	Male	103	Male	203	Female	303	Male	3	Female
4	Male	104	Female	204	Female	304	Female	4	Male
5	Female	105	Male	205	Male	305	Male	5	Male
6	Female	106	Female	206	Male	306	Male	6	Female
7	Male	107	Female	207	Female	307	Male	7	Male
8	Female	108	Male	208	Male	308	Male	8	Female
9	Female	109	Male	209	Female	309	Female	9	Male
10	Male	110	Female	210	Female	310	Female	10	Female
11	Male	111	Female	211	Male	311	Male	11	Male
12	Female	112	Male	212	Male	312	Male	12	Female
13	Male	113	Male	213	Male	313	Female	13	Female
14	Male	114	Male	214	Female	314	Female	14	Male
15	Male	115	Female	215	Female	315	Male	15	Male
16	Male	116	Male	216	Male	316	Female		
17	Male	117	Male	217	Male	317	Male		
18	Male	118	Male	218	Male	318	Female		
19	Male	119	Male	219	Male	319	Male		
20	Male	120	Male	220	Male	320	Male		
21	Female	121	Male	221	Male	321	Female		
22	Male	122	Male	222	Male	322	Male		

23	Male	123	Male	223	Female	323	Male
24	Male	124	Female	224	Male	324	Male
25	Male	125	Male	225	Female	325	Female
26	Male	126	Male	226	Male	326	Female
27	Female	127	Male	227	Male	327	Female
28	Female	128	Male	228	Male	328	Female
29	Female	129	Male	229	Male	329	Female
30	Male	130	Male	230	Male	330	Female
31	Male	131	Male	231	Male	331	Male
32	Male	132	Female	232	Male	332	Male
33	Female	133	Female	233	Female	333	Female
34	Male	134	Male	234	Male	334	Female
35	Male	135	Male	235	Male	335	Male
36	Male	136	Female	236	Female	336	Male
37	Male	137	Female	237	Female	337	Male
38	Female	138	Female	238	Male	338	Male
39	Male	139	Male	239	Male	339	Male
40	Female	140	Female	240	Female	340	Male
41	Female	141	Female	241	Male	341	Male
42	Male	142	Male	242	Female	342	Female
43	Male	143	Female	243	Male	343	Male
44	Male	144	Female	244	Female	344	Male
45	Female	145	Female	245	Male	345	Male
46	Female	146	Male	246	Male	346	Female
47	Female	147	Male	247	Male	347	Male
48	Male	148	Male	248	Female	348	Male
49	Male	149	Female	249	Female	349	Male
50	Female	150	Male	250	Male	350	Male
51	Female	151	Male	251	Male	351	Female
52	Female	152	Female	252	Male	352	Male
53	Male	153	Male	253	Male	353	Male
54	Female	154	Male	254	Male	354	Female
55	Female	155	Male	255	Female	355	Female
56	Female	156	Female	256	Male	356	Female
57	Male	157	Female	257	Male	357	Male
58	Male	158	Female	258	Female	358	Male
59	Male	159	Female	259	Male	359	Female
60	Female	160	Female	260	Male	360	Male
61	Male	161	Male	261	Male	361	Male
62	Female	162	Female	262	Male	362	Male
63	Female	163	Female	263	Male	363	Female
64	Male	164	Male	264	Male	364	Female
65	Female	165	Female	265	Female	365	Male
66	Female	166	Male	266	Male	366	Female

67	Male	167	Male	267	Female	367	Male
68	Male	168	Female	268	Female	368	Female
69	Female	169	Male	269	Male	369	Male
70	Male	170	Male	270	Female	370	Female
71	Female	171	Male	271	Female	371	Male
72	Female	172	Female	272	Male	372	Female
73	Female	173	Female	273	Male	373	Female
74	Female	174	Male	274	Male	374	Female
75	Female	175	Male	275	Male	375	Female
76	Female	176	Female	276	Female	376	Female
77	Male	177	Male	277	Female	377	Male
78	Male	178	Male	278	Male	378	Male
79	Female	179	Male	279	Male	379	Female
80	Male	180	Female	280	Female	380	Male
81	Male	181	Female	281	Male	381	Female
82	Female	182	Female	282	Female	382	Female
83	Male	183	Female	283	Male	383	Male
84	Female	184	Female	284	Female	384	Male
85	Female	185	Female	285	Female	385	Female
86	Male	186	Female	286	Female	386	Male
87	Male	187	Male	287	Female	387	Male
88	Male	188	Male	288	Male	388	Male
89	Male	189	Male	289	Male	389	Female
90	Male	190	Male	290	Female	390	Male
91	Male	191	Male	291	Male	391	Male
92	Male	192	Female	292	Female	392	Male
93	Male	193	Male	293	Female		
94	Male	194	Female	294	Female		
95	Male	195	Male	295	Female		
96	Male	196	Male	296	Male		
97	Male	197	Male	297	Male		
98	Female	198	Male	298	Male		
99	Female	199	Male	299	Female		
100	Male	200	Male	300	Female		

Supplementary Table 3 Feed formula for AA feeding

	Control	1.5% AA
Casein	205	205
Corn starch	368	368
Dextrin	135	135
Sucrose	102	102
Soybean oil	72	72

Cellulose	51	51
Minerals	36	36
Vitamin	10	10
L-cystine	3	3
Choline tartrate	3	3
TBHQ	0.01	0.01
Cocoa butter	15	0
Arachidonic acid	0	15
Total(g)	1000	1000

Supplementary materials and methods

Patients and samples

A total of 392 paraffin sections of CRC tissues and adjacent paired non-cancerous tissues were collected from the Department of Gastrointestinal Surgery, Renji Hospital, School of Medicine, Shanghai Jiao Tong University. These sections were used to design a tissue microarray. All patients with CRC underwent surgery at the Department of Gastrointestinal Surgery, Renji Hospital, School of Medicine, Shanghai Jiao Tong University between January 2014 and January 2016. The matched CRC, adenoma, and non-cancerous tissues were collected from 15 CRC cases and stored −80°C. The gender of patients showed in Supplementary Table 2. The study was approved by the Research Ethics Committee of Renji Hospital (2018-064) and carried out under the ethical standards formulated in the Helsinki Declaration. Informed consent was provided by all patients.

Cell culture

SW480 (SCSP-5033), HCT116 (TCHu 99), SW620 (TCHu101), HT29 (SCSP-5032), LoVo (TCHu 82), RKO (TCHu116) cells (human CRC cell lines) were obtained from the Cell Bank of the Chinese Academy of Sciences (Shanghai, China), and NCM460 cells (human intestinal epithelial cell line, JZ-006820) were obtained from the Juzhou Biotechnology Co., LTD (Anhui, China) , which all performed genotyping of human cancer cell lines by short tandem repeat (STR) profiling and had no cross-contamination. All cell lines were cultured in Dulbecco's modified Eagle's medium supplemented with 10% fetal bovine serum (10099, Gibco) and 1% penicillin and streptomycin at 37°C with 5% CO2 in a humidified incubator. To further confirm the role of AA, fatty acid free fetal bovine serum (10%) was used to cell culture (Fetal Bovine Serum (Australia), Lipid Depleted, S1370L, BIOAGRIO).

Lentivirus and siRNA transfection

An appropriate amount of CRC cells were added and cultured in 6-well plates, until the cells grew to about 60% in each well. siRNA and shRNA transfection were performed. siRNA transfection: two sterile 1.5ml EP tubes were taken, one was supplemented with 250ul Opti-MEM medium (31985070, Gibco™) and 5ul Lipofectamine™ RNAiMAX Transfection Reagent (13778057, Thermo Fisher), and the other was supplemented with 250ul Opti-MEM medium and 5ul siRNA fragment. Mix well and

stand for 5 minutes, respectively. Then mix the two tubes and stand for 20 minutes. The mixed solution was added to each well of the 6-well plate, and 1ml 10% FBS of DMEM medium without 1% penicillin and streptomycin was added and cultured for 12 hours, then the medium was changed to 10% FBS of DMEM medium with 1% penicillin and streptomycin. After 48 hours, the interference efficiency was detected by q-PCR and western blot. siNC used as a negative control. Lentivirus transfection: the lentivirus with shRNAs (10^8 TU/ml) was constructed and obtained from GenePharma (Shanghai GenePharma Co., Ltd., Shanghai, China). A sterile 1.5ml EP tube was taken and added with 100ul lentivirus liquid, 1.5 ml Opti-MEM and 2μg/ml polybrene. Mix well and add to to each well of the 6-well plate. After 24h, the medium was changed to 10% FBS of DMEM medium with 1% penicillin and streptomycin. When the transfected CRC cells covered the entire 6-well plate, the puromycin was added to screen the successfully transfected cells, the screening cycle was 2 weeks. The interference efficiency was detected by q-PCR and western blot. shNC used as a negative control.

Reagents

Azoxymethane was purchased from Sigma-Aldrich (A5486). Dextran Sulfate Sodium was purchased from MP Biomedicals (9011-18-1). Arachidonic acid was purchased from Sigma-Aldrich (A3611).

Lipopolysaccharide was purchased from Sigma-Aldrich (L4391). TLR4 inhibitor, TAK-242, and PTGS2 inhibitor, celecoxib, were purchased from Selleck (S7455 and SC58635), TAK-242 was intraperitoneal injection with 3mg/kg and celecoxib was suspended in 0.5% methylcellulose and orally treated at 100mg/kg to the tumor-bearing mice every day. FADS1 inhibitor, D5D-IN-326 (2-(2,2,3,3,3-pentafluoropropoxy)-3-[4- (2,2,2-trifluoroethoxy)phenyl]-5,7-dihydro-3H-pyrrolo[2,3-d]pyrimidine-4,6-dione), was purchased from MedChemExpress (HY-117427) and orally treated at 2mg/kg for 2 weeks. Proteinase K (G1205), DAPI (G1012) and EdU assay kit (G1501) was purchased from Servicebio.

Animal experiments

AOM/DSS-induced CRC: Male C57BL/6J mice (4 weeks of age) were purchased from the Animal Room, East China Normal University (Shanghai, China) and raised in an SPF animal room. After two weeks of adaptive feeding, a single intraperitoneal injection of azoxymethane (AOM, 10 mg/kg), 2% dextran sodium sulfate (DSS) (1 week) and normal water intake (1 week) for 3 cycles were used to induce CRC. Tumor tissues were collected and a portion stored at −80°C. The remaining tumor tissues were embedded in paraffin for histological examination. At antibiotic treatment groups, mice were pretreated with

quadruple antibiotics for two weeks, including ampicillin (0.2 g/L), vancomycin (0.1 g/L), neomycin (0.2 g/L), and metronidazole (0.2 g/L) to eliminate intestinal flora before AOM/DSS treatment. For selective antibiotic treatment experiment, aztreonam (0.2 g/L) was used to eliminate Gram-negative bacteria and vancomycin (0.1 g/L) was used to eliminate Gram-positive bacteria for two weeks before AOM/DSS treatment.

Spontaneous CRC model: *Apc*$^{flox/flox}$ mice and *Lgr5-EGFP-IRES-CreERT2* mice were obtained from the Animal Room, East China Normal University (Shanghai, China). All the above mice were on the C57BL/6 genetic background. *Apc*$^{flox/flox}$ mice were crossed with *Lgr5-EGFP-IRES-CreERT2* mice for ablation of *Apc* expression in Lgr5+ cells was deleted with four consecutive daily doses of Tamoxifen (50 mg/kg). RNA was identified five days after the initial dose by quantitative reverse transcription PCR (qRT-PCR). 20-24 weeks intestinal specific *Apc*$^{-/-}$ mice were used in this article. Antibiotic treatment was consistent with AOM/DSS mice.

Mice feeding: AIN-93G diet was used for the control diet (NC feeding), diet enriched with AA-FFA 1.5% for AA feeding, and 2 diets were isocaloric. In order to prevent AA from being oxidized, the feed is

vacuum-packed (50g/per pack), stored at low temperature (4°C) and avoided repeated use. All feeds are daily provided. The nutritional composition of the mice feed are shown in Supplementary Table 3.

Table S2 Feed formula for mice

	Control	1.5% AA
Casein	205	205
Corn starch	368	368
Dextrin	135	135
Sucrose	102	102
Soybean oil	72	72
Cellulose	51	51
Minerals	36	36
Vitamin	10	10
L-cystine	3	3
Choline tartrate	3	3
TBHQ	0.01	0.01
Cocoa butter	15	0
Arachidonic acid	0	15
Total(g)	1000	1000

Fecal collection and fecal transplant: Male C57BL/6J mice (4 weeks of age) were purchased and raised in an SPF animal room. After two weeks of adaptive feeding, the mice were randomly divided into two groups, including AA feeding and NC feeding groups. After 12 weeks, mouse feces of each group were collected by sterile instruments for gut microbiota analysis and fecal transplant. AOM/DSS and *Apc*$^{-/-}$ Mice that received fecal transplants were pretreated with quadruple antibiotics for two weeks, including ampicillin (0.2 g/L), vancomycin (0.1 g/L), neomycin (0.2 g/L), and metronidazole (0.2 g/L) to eliminate intestinal

flora. Mice were divided into stool-AA (stool from AA feeding) and stool-NC (stool from normal feeding) groups and administered 200ul stool suspension by gavage (twice a week). One gram of mixed stool was then suspended in 5 mL PBS.

Xenograft model： Athymic male null mice (6 weeks) were used for the subcutaneous xenograft model, purchased from the Animal Room, East China Normal University (Shanghai, China). All the above mice were on the BALB/C genetic background. Five million SW480 or HCT116 cells were injected into left anterior flank of each null mouse. Tumor diameters were monitored with callipers every week of mice for 4 weeks. After 4 weeks, all null mice were sacrificed and xenograft tumors excised. Part of the tissues were fixed in 4% paraformaldehyde for histological analysis. The other tissues were stored at −80° C. Tumor volume and weight were recorded. Tumor volume was calculated wath length*width^2/2. The reagent treatments (intratumor injection) were as follows: AA (2mg/L), LPS (1 μg/ml), PGE2 (1mg/L). 100ul reagents were injected into subcutaneous xenograft once a week.

Orthotopic model: Nude mice (6 weeks, male, BALB/C) were anesthetized with 0.5% pentobarbital. After opening the abdominal cavity, 5 × 10^6 SW480^{Luc} or HCT116^{Luc} cells were injected into the ileocecum. Mice bearing orthotopic tumor were intraperitoneally injected with

150mg/200ul D-luciferin, anaesthetized with 2.5% vapourized inhaled isoflurane, placed into an IVIS Spectrum imaging system (Perkin Elmer Lifelmage 4.5.5) (Caliper Life Sciences, Waltham, MA). Computed tomography (CT) was then performed to merge with firefly bioluminescence signals. For organ reconstruction, CT images were placed combined with the autoreconstructed intestine and colon at an opacity of 20%. The normalization and quantification of firefly bioluminescence signals were performed according to the surrounding area of the red cube. After 4 weeks, mice were sacrificed and tumor tissues were excised. Part of the tissues were fixed in 4% paraformaldehyde for histological analysis. The other tissues were stored at −80°C. At antibiotic treatment groups, mice were pretreated with quadruple antibiotics for two weeks, including ampicillin (0.2 g/L), vancomycin (0.1 g/L), neomycin (0.2 g/L), and metronidazole (0.2 g/L) to eliminate intestinal flora. For selective antibiotic treatment experiment, aztreonam (0.2 g/L) was used to eliminate Gram-negative bacteria and vancomycin (0.1 g/L) was used to eliminate Gram-positive bacteria for two weeks in nude mice.

Organoid model: Intestinal tumor tissue was obtained from AOM/DSS mice. Tumor tissue was shredded on ice and digested with digestive liquid for 30min (digestion on a constant temperature culture shaker).

After terminating digestion, supernatant was removed by centrifugation at 500g for 5min. After re-suspension by PBS, the supernatant was filtered through 70μm filter, remove supernatant after centrifugation, add 200ul organoid *in vitro* medium and fully mix. Add pre-thawed Matrigel matrix glue to equal ratio mixture, take 50ul of the mixture and add it to the middle part of the 24-well plate that has been preheated for 30min, put the 24-well plate into the incubator and stand for 20min, add 500ul organoid in vitro medium after the gel solidified, and add PBS to the other holes to ensure humidity. Organoid cells were transfected with a firefly luciferase before Lentivirus transfection to knockdown Fads1. Then a orthotopic model was conducted with Male C57BL/6J mice (4 weeks of age). Mice were anesthetized with 0.5% pentobarbital. After opening the abdominal cavity, 5×10^6 organoid cells were injected into the ileocecum. The orthotopic tumor was detected by IVIS Spectrum imaging system. After 4 weeks, mice were sacrificed and tumor tissues were excised. Part of the tissues were fixed in 4% paraformaldehyde for histological analysis. The other tissues were stored at −80°C. Antibiotic treatments were consistent with orthotopic model.

Liquid Chromatography Mass Spectrometry

20 mg of tumor tissue was accurately weighed, 200μL 10% methanol (including BHT) was added, vortex mixing, ultrasound in ice bath for 30

min, arachidonic acid substances were extracted by HLB μelution system, 200μL methanol activation, 200μL water equilibrium, sample loading. Rinse with 200 μL water, then rinse with 200μL 10% methanol solution, 50μL acetonitrile aqueous solution elution (acetonitrile: water: formic acid = 80:20:0.02; v/v/v). The samples were separated by Agilent 1290 Infinity LC liquid chromatography system. A 5500 QTRAP mass spectrometer (AB SCIEX) was used for mass spectrometry analysis in negative ion mode.The peak area and retention time were extracted by Multiquant software. The standard arachidonic acid was used to correct the retention time and identify the metabolites.

RNA isolation and real-time quantitative polymerase chain reaction (RT-qPCR)

Real-time PCR was also used to detection of *Es. coli*, *En. faecalis* and *Lactobacillus*. *Es.coli* primers:F: 5′-CAATTTTCGTGTCCCCTTCG-3′; R:5′-GTTAATGATAGTGTGTCGAAAC-3′.*En.faecalis*:F:5′-CCGAGTG CTTGCACTCAATTGG-3′;R:5′-CTCTTATGCCATGCGGCATAAAC-3′. *Lactobacillus*:F:5′-CTGATGAAAGCCCTCG-3′;R:5′-GAGCCTCAGCG TCAGTTG-3′. Actin as an internal control, F:5′-AAAAGCCACCCCACTTCTCT-3′;R:5′-CTCAAGTTGGGGGAC AAAAA-3′. Primer for total bacteria detection:F:5′-GTGSTGCAYGGYTGTCGTCA-3′;R:5′-ACGTCRTCCM

CACCTTCCTC-3′, Relative expression of target genes was calculated by the $2^{-\triangle\triangle}$Ct method.

Protein extraction and western blotting

Total protein was extracted using radioimmunoprecipitation assay (RIPA) buffer supplemented with 1% protease and phosphatase inhibitors (78440, Thermo Fisher Scientific). Protein concentration was measured by bicinchoninic acid assay with a BCA Protein Assay Kit (Pierce Biotechnology) and keep a unified concentration. 5x SDS-PAGE Sample Loading Buffer (Beyotime; P0015) was added to protein samples and cooked in boiling water for 10min. Put 8-12% SDS−PAGE gel into the electrophoresis tank with enough electrophoresis solution. Add 20ul sample to each well and then perform protein electrophoresis (80V, 30min and 120V, 60min). Then, the protein in the SDS−PAGE gel was transferred onto NC membranes. skimmed milk powder (Invitrogen) diluted in TBST (containing 1‰ Tween 20) at a concentration of 5% was used to block non-specific binding for 1-2 hours at room temperature. Primary antibodies were FADS1 (ab126706, Abcam, 1:000), TLR4 (ab13556, Abcam, 1:1000), β-actin (ab8227, Abcam, 1:1000), MYD88 (ab28763, Abcam, 1:1000), PTGES (ab233274, Abcam, 1:1000), PTGS2 (ab179800, Abcam, 1:1000), proliferating cell nuclear antigen (10205-2-AP, Proteintech Group, Inc., 1:1000). Secondary antibodies,

horseradish peroxidase (HRP)-conjugated Affinipure Goat Anti-Rabbit IgG (H+L) (SA00001-2, 1:1000) and HRP-conjugated and Affinipure Goat Anti-Mouse IgG (H+L) (SA00004-1, 1:1000), were obtained from Proteintech Group, Inc. Overnight incubation with primary antibodies and incubation with secondary antibodies for 1h. All the bands were treated with Lumi Q ECL reagent solution kit (SB-WB012, Share-Bio) and detected by a ChemiDocTM XRS+ system (BIO-RAD).

Cell proliferation

Cell proliferation was assessed using a Cell Counting Kit-8 (CCK8, Dojindo, Japan) following kit instructions. 3000/well CRC cells were seeded into 96-well plates. add PBS to the other holes to ensure humidity. Every 24h, the numbers of viable cells were measured using a microplate reader (BioTek, US) at an absorbance of 450 nm. The duration of testing was 4-5 days. At detection time point, 100 μl of diluted CCK-8 reagent in culture medium at a concentration of 10% (v/v) was added to every well and incubated with cells at 37°C for 1 hour. The data of CCK-8 was detected by using Thermo Scientific Multiskan FC. For each group, 5 replicate wells were prepared and all experiments were replicated independently three times. The reagent treatment is as follows: AA (0.5mg/L, 1mg/L, 2mg/L), LPS (1 μg/ml), PGE2 (1mg/L).

Colony formation analysis

1000/well CRC cells were seeded into 6-well plates. The medium was changed every 3 days. After two weeks, cells were fixed in 4% paraformaldehyde for 30min and stained with 0.1% crystal violet solution, and the numbers of colonies (>50 cells) were counted under a microscope. Each experiment was carried out independently in triplicate. The reagent treatment is as follows: AA (0.5mg/L, 1mg/L, 2mg/L), LPS (1 μg/ml), PGE2 (1mg/L).

EdU assay

A Click-iT EdU kit (C10337, Thermo) was used following kit instructions. CRC cells were seeded into 12-well plates, 2×10^4 cells/well. After 48h, cells were incubated with 50 μM EdU buffer, fixed with 4% formaldehyde, and permeabilized with 0.1% Triton X-100. EdU solution was added then add followed by staining of nuclei with Hoechst. Each experiment was carried out independently in triplicate. The reagent treatment is as follows: AA (0.5mg/L, 1mg/L, 2mg/L). Confocal microscopy images were taken using Leica SP8 LAS X.

Elisa assay

Tumor tissue: fresh tumor tissue was used, cut and weighed 100mg, frozen quickly with liquid nitrogen, ground the tissue manually, added

1ml PBS, and homogenized further with ultrasonic cell crusher.

CRC cells: 10^7 cells/sample were obtained and digested, 0.2ml PBS was added and repeated freeze-thaw with liquid nitrogen, and homogenized further with ultrasonic cell crusher. All the samples was centrifuged for 10 minutes (1000 g) and collected the supernatant.

Interstitial fluid: 300mg fresh tumor tissues were supported with triple-layered 10μm nylon mesh in the tube and centrifuged at 28 g for 5 minutes to remove surface liquids of tissues, followed by centrifugation at 1000 g for another 10 minutes to collect interstitial fluids.

AA, EPA, and PGE2 were measured by detection kits (AA:NBP2-59872, Novus Biologicals; EPA:LM-11519-ES, Lianmai Biological Technology Co., Ltd.; PGE2: KGE004B, Novus Biologicals), respectively. In brief, according to instructions, detection procedure included adding standard samples, adding test samples, adding enzymes, incubation at 37°C for 60 min, washing, coloring, stopping the reaction and measurement by using Thermo Scientific Multiskan FC.

Microarray data analysis

GDS4382 and GSE41657 were searched and downloaded from the GEO database (https://www.ncbi.nlm.nih.gov/geo/). The COAD and READ dataset was searched and downloaded from the TCGA dataset. The analysis of differentially expressed genes (DEGs) used both fold change

and the student’ s *t*-tests. Fold change (FC)>2 and p-value <0.05 were deemed statistically significant. DEGs were used for KEGG pathway enrichment with a threshold for statistical significance of $p < 0.05$.

Statistical analysis

All measurement data were were evaluated with two-way tudent’s *t*-tests. Correlation of measurement data was analyzed by Pearson correlation. Relationship between the FADS1 expression with clinical variables in CRC patients was evaluated with different statistical methods. Data such as age and tumor size were evaluated with Student’s *t*-tests. Categorical variables, such as sex, T stage, lymph node invasion, and distant metastasis, were analyzed via chi square or Fisher's exact test. Rank sum test was used for the analysis of two-way ordered categorical data. Survival curves were generated using the Kaplan–Meier method and analyzed with log-rank tests.
