# Supplementary material for: FADS1-arachidonic acid axis enhances arachidonic acid metabolism by altering intestinal microecology in colorectal cancer
Source: Nat Commun. 2023 Apr 11;14:2042. doi: 10.1038/s41467-023-37590-x (PMC10090135; doi:10.1038/s41467-023-37590-x)
Supplement: Supplementary file 3 — Reporting Summary [file 41467_2023_37590_MOESM3_ESM.pdf]

nature portfolio

Corresponding author(s): Qing Xu, Xue-li Zhang, Zhi-gang Zhang,Jun Li, Han-bing Xue

Last updated by author(s): Feb 7, 2023

Reporting Summary

Nature Portfolio wishes to improve the reproducibility of the work that we publish. This form provides structure for consistency and transparency in reporting. For further information on Nature Portfolio policies, see our Editorial Policies and the Editorial Policy Checklist.

Statistics

For all statistical analyses, confirm that the following items are present in the figure legend, table legend, main text, or Methods section.

n/a	Confirmed	
☐	☒	The exact sample size (*n*) for each experimental group/condition, given as a discrete number and unit of measurement
☐	☒	A statement on whether measurements were taken from distinct samples or whether the same sample was measured repeatedly
☐	☒	The statistical test(s) used AND whether they are one- or two-sided<br>*Only common tests should be described solely by name; describe more complex techniques in the Methods section.*
☒	☐	A description of all covariates tested
☐	☒	A description of any assumptions or corrections, such as tests of normality and adjustment for multiple comparisons
☐	☒	A full description of the statistical parameters including central tendency (e.g. means) or other basic estimates (e.g. regression coefficient) AND variation (e.g. standard deviation) or associated estimates of uncertainty (e.g. confidence intervals)
☐	☒	For null hypothesis testing, the test statistic (e.g. F, t, r) with confidence intervals, effect sizes, degrees of freedom and P value noted<br>*Give P values as exact values whenever suitable.*
☒	☐	For Bayesian analysis, information on the choice of priors and Markov chain Monte Carlo settings
☒	☐	For hierarchical and complex designs, identification of the appropriate level for tests and full reporting of outcomes
☐	☒	Estimates of effect sizes (e.g. Cohen's *d*, Pearson's *r*), indicating how they were calculated

Our web collection on statistics for biologists contains articles on many of the points above.

Software and code

Policy information about availability of computer code

Data collection	Confocal microscopy images were taken using Leica SP8 LAS X. Pathological images were acquired using Leica Aperio ScanScope Console 12.3. CCK8 and Elisa data were acquired using Thermo Scientific Multiskan FC. 16S RNA sequencing data were collected using HiSeq 2500. Luciferin emission imaging was performed with Perkin Elmer Lifelmage 4.5.5. 5500 QTRAP mass spectrometer (AB SCIEX) was used for mass spectrometry analysis
Data analysis	RNA sequencing data were analyzed with R-3.3.1. For statistical analysis and graphical data presentation we used GraphPad Prism 7 and SPSS20.0.

For manuscripts utilizing custom algorithms or software that are central to the research but not yet described in published literature, software must be made available to editors and reviewers. We strongly encourage code deposition in a community repository (e.g. GitHub). See the Nature Portfolio guidelines for submitting code & software for further information.

Data

Policy information about availability of data

All manuscripts must include a data availability statement. This statement should provide the following information, where applicable:

- Accession codes, unique identifiers, or web links for publicly available datasets
- A description of any restrictions on data availability
- For clinical datasets or third party data, please ensure that the statement adheres to our policy

The 16S rRNA sequence data generated in this study were deposited in the NCBI Sequence Read Archive (SRA) database under the accession code PRJNA762520 (https://www.ncbi.nlm.nih.gov/bioproject/PRJNA76 2520). Public data used in this work can be acquired from the TCGA Research Network portal (https://portal.gdc.cancer.gov/projects/TCGA-COAD; https://portal.gdc.cancer.gov/projects/TCGA-READ) and Gene Expression Omnibus (GDS4382 and GSE41657, (https://www.ncbi.nl m.nih.gov/geo/query/acc.cgi?acc=GDS4382; https://www.ncbi.nlm.nih. gov/geo/query/acc.cgi?acc=GSE41657). All of the source data in this study are provided with this paper as Source data file.

Human research participants

Policy information about studies involving human research participants and Sex and Gender in Research.

Reporting on sex and gender	These information could be available on Supplementary Table 2
Population characteristics	The detailed participants characteristics such as age and gender could be available on Supplementary Table 2
Recruitment	CRC tissues and adjacent paired non-cancerous tissues were collected from the Department of Gastrointestinal Surgery, Renji Hospital, School of Medicine, Shanghai Jiao Tong University. All patients with CRC underwent surgery at the Department of Gastrointestinal Surgery, Renji Hospital, School of Medicine, Shanghai Jiao Tong University between January 2014 and January 2016.
Ethics oversight	The study was approved by the Research Ethics Committee of Renji Hospital (2018-064) and carried out under the ethical standards formulated in the Helsinki Declaration. Informed consent was provided by all patients.

Note that full information on the approval of the study protocol must also be provided in the manuscript.

Field-specific reporting

Please select the one below that is the best fit for your research. If you are not sure, read the appropriate sections before making your selection.

[x] Life sciences [ ] Behavioural & social sciences [ ] Ecological, evolutionary & environmental sciences

For a reference copy of the document with all sections, see nature.com/documents/nr-reporting-summary-flat.pdf

Life sciences study design

All studies must disclose on these points even when the disclosure is negative.

Sample size	The sample size is not predetermined by statistical methods, but is based on the expected effect size and variability of the sample size, as well as the cost and feasibility of the experiment.
Data exclusions	No data were excluded from analysis in our study
Replication	Experiments in the article are reliably produced, replication were described in the figure legends.
Randomization	All mice and cells were randomly assigned to experimental groups.
Blinding	Investigators were blinded for most of the qualification experiments. Also, quantification of tumor parameters by histological analyses was performed in a blinded fashion.

Reporting for specific materials, systems and methods

We require information from authors about some types of materials, experimental systems and methods used in many studies. Here, indicate whether each material, system or method listed is relevant to your study. If you are not sure if a list item applies to your research, read the appropriate section before selecting a response.

Materials & experimental systems

n/a	Involved in the study	
☐	☒	Antibodies
☐	☒	Eukaryotic cell lines
☒	☐	Palaeontology and archaeology
☐	☒	Animals and other organisms
☒	☐	Clinical data
☒	☐	Dual use research of concern

Methods

n/a	Involved in the study	
☒	☐	ChIP-seq
☒	☐	Flow cytometry
☒	☐	MRI-based neuroimaging

Antibodies

Antibodies used

Primary antibodies were FADS1 (ab126706, Abcam, 1:000), TLR4 (ab13556, Abcam, 1:1000), β-actin (ab8227, Abcam, 1:1000), MYD88 (ab28763, Abcam, 1:1000), PTGES (ab233274, Abcam, 1:1000), PTGS2 (ab179800, Abcam, 1:1000), proliferating cell nuclear antigen (Proteintech Group, Inc., 1:1000). Secondary antibodies, horseradish peroxidase (HRP)-conjugated Affinipure Goat Anti-Rabbit IgG (H+L) (SA00001-2, 1:1000) and HRP-conjugated and Affinipure Goat Anti-Mouse IgG (H+L) (SA00004-1, 1:1000), were obtained from Proteintech Group, Inc.

Validation

All the antibodies involved in this study were selected and utilized based on manufactures' validations. Antibody validation information can be found on manufacturers' website:FADS1:https://www.abcam.cn/fads1-antibody-epr6898-ab126706.html; TLR4:https://www.abcam.cn/tlr4-antibody-ab13556.html; β-actin:https://www.abcam.cn/beta-actin-antibody-ab8227.html; MYD88:https://www.abcam.cn/myd88-antibody-ab28763.html;
PTGES:https://www.abcam.cn/ptges2gbf1-antibody-ab233274.html;
PTGS2:https://www.abcam.cn/cox2--cyclooxygenase-2-antibody-epr12012-ab179800.html;
PCNA:https://www.ptgcn.com/products/PCNA-Antibody-10205-2-AP.htm;
Horseradish peroxidase (HRP)-conjugated Affinipure Goat Anti-Rabbit IgG (H+L): https://www.ptgcn.com/products/HRP-conjugated-Affinipure-Goat-Anti-Rabbit-IgG-H-L-secondary-antibody.htm;
HRP-conjugated and Affinipure Goat Anti-Mouse IgG (H+L): https://www.ptgcn.com/products/Biotin-conjugated-Affinipure-Goat-Anti-Mouse-IgG-H-L-secondary-antibody.htm

Eukaryotic cell lines

Policy information about cell lines and Sex and Gender in Research

Cell line source(s)

SW480 (SCSP-5033), HCT116 (TCHu 99), SW620 (TCHu101), HT29 (SCSP-5032), LoVo (TCHu 82), RKO (TCHu116) cells (human CRC cell lines) were obtained from the Cell Bank of the Chinese Academy of Sciences (Shanghai, China), and NCM460 cells (human intestinal epithelial cell line, JZ-006820) were obtained from the Juzhou Biotechnology Co., LTD (Anhui, China)

Authentication

All cell lines were validated using short tandem repeat (STR) profiling.

Mycoplasma contamination

All the cell lines had no mycoplasma contamination

Commonly misidentified lines
(See ICLAC register)

No commonly misidentified cell lines were used in this study.

Animals and other research organisms

Policy information about studies involving animals; ARRIVE guidelines recommended for reporting animal research, and Sex and Gender in Research

Laboratory animals

Spontaneous CRC model: Apcflox/flox mice and Lgr5-EGFP-IRES-CreERT2 mice were obtained from the Animal Room, East China Normal University (Shanghai, China). 20-24 weeks intestinal specific Apc-/- mice were used in this article. All the above mice were on the C57BL/6 genetic background.
AOM/DSS-induced CRC: Male C57BL/6J mice (4 weeks of age) were purchased from the Animal Room, East China Normal University (Shanghai, China) and carried out a AOM/DSS induced assay during 12 weeks.
Xenograft model and Orthotopic model: Athymic male null mice (6 weeks) were used for the subcutaneous xenograft model, purchased from the Animal Room, East China Normal University (Shanghai, China). All the above mice were on the BALB/C genetic background.
Organoid model: orthotopic model was conducted with Male C57BL/6J mice (4 weeks of age)
Animals were housed in East China Normal University SPF animal facility, in temperatures 20-22℃, humidity 30-70% and a 12-hour light/12-hour dark cycle.

Wild animals

This study did not involve wild animals.

Reporting on sex

For spontaneous CRC model, Equal ratio of male and female. For AOM/DSS-induced CRC model, Xenograft model , Orthotopic model and Organoid model, male mice was used because that male mice was more stronger and less susceptible to hormone levels.

Field-collected samples	No field-collected samples were used in the study.
Ethics oversight	All animal experiments were approved by the Research Ethics Committee of Renji Hospital and East China Normal University and adhered to local and national requirements for the care and use of laboratory animals.

Note that full information on the approval of the study protocol must also be provided in the manuscript.
